# A Phase I, Open-Label, Dose Escalation Study of Enoblituzumab in Children and Young Adults with B7-H3–Expressing Relapsed or Refractory Solid Tumors

**DOI:** 10.1158/2767-9764.CRC-25-0293

**Published:** 2025-09-10

**Authors:** Kenneth B. DeSantes, Kimberly A. McDowell, Paul M. Sondel, Paul R. Hutson, Rosandra N. Kaplan, Julie R. Park, Meenakshi G. Hegde, Crystal L. Mackall, John M. Maris

**Affiliations:** 1 Division of Hematology/Oncology, Department of Pediatrics, University of Wisconsin – American Family Children’s Hospital, Madison, Wisconsin.; 2 University of Wisconsin School of Pharmacy, UW Carbone Comprehensive Cancer Center, Madison, Wisconsin.; 3 Pediatric Oncology Branch, National Cancer Institute, Center for Cancer Research, Bethesda, Maryland.; 4 Divison of Hematology, Oncology, Bone Marrow Transplant and Cellular Therapy, Seattle Children's Hospital, Seattle, Washington.; 5 Texas Children’s Cancer Center, Department of Pediatrics, Baylor College of Medicine, Houston, Texas.; 6 Division of Hematology/Oncology, Department of Pediatrics, Stanford Medicine and Children’s Health, Palo Alto, California.; 7 Division of Oncology, Children’s Hospital of Philadelphia, Philadelphia, Pennsylvania.

## Abstract

**Purpose:**

This multicenter, phase I, cohort expansion study was performed to characterize the safety, pharmacokinetics, pharmacodynamics, immunogenicity, and preliminary antitumor activity of enoblituzumab in children with relapsed/refractory tumors expressing B7-H3 above a predetermined threshold.

**Patients and Methods:**

Samples from 101 patients were screened for B7-H3 expression. Twenty-five patients with relapsed/refractory B7-H3-expressing solid tumors were enrolled in the trial with a median age of 14.8 years (range, 6–24 years). Enoblituzumab was administered intravenously weekly at 10 or 15 mg/kg.

**Results:**

B7-H3 expression was documented in 96% of evaluable tumors by IHC. The maximum administered dose of enoblituzumab was the protocol-specified maximum dose of 15 mg/kg as no dose-limiting toxicities were observed. Patients received a median of 6.5 doses (range 1–24), and the main toxicities encountered were infusion-related reactions. Pharmacokinetic studies showed that drug plasma concentrations fit a linear two-compartment model with a mean elimination half-life of 67 days. No objective tumor responses were documented.

**Conclusions:**

B7-H3 is expressed on a high fraction of pediatric solid tumors spanning many different disease histologies. Enoblituzumab was well tolerated at a weekly dose of 15 mg/kg and induced inflammatory reactions in some patients though no objective responses were observed. Further testing of enoblituzumab in combination with other agents, or the use of other B7-H3–directed therapeutics, should be considered for children with relapsed solid tumors.

**Significance:**

This phase I clinical trial is the first study to evaluate the feasibility and safety of administering enoblituzumab to pediatric patients with relapsed solid tumors. We demonstrated that B7-H3 is highly expressed across a wide variety of tumor histologies. Enoblituzumab could be safely administered at a dose of 15 mg/kg. However, no objective responses were observed, suggesting that alternative strategies to target B7-H3 for children with relapsed tumors should be considered.

## Introduction

Although significant progress has been made in recent years in the clinical outcomes for many pediatric cancers, the prognosis for children with relapsed or refractory solid tumors remains dismal. There is clearly a need to develop new treatments for these patients. B7-H3 (encoded by the *CD276* gene) is a member of the B7 family of immune regulatory cell surface molecules. Compared with normal tissues, B7-H3 is overexpressed in a variety of pediatric solid tumors, including neuroblastoma (1–5), rhabdomyosarcoma (3, 5–7), osteosarcoma (5, 8), Ewing sarcoma (5, 6), Wilms tumor (5), and desmoplastic small round cell tumor (4, 5), as well as in a number of cancers more common in adults (9–13). In certain malignancies, B7-H3 is also expressed on tumor vasculature and has been associated with metastatic behavior and poor clinical outcomes (10, 11, 14). Thus, B7-H3 is an attractive target for cancer immunotherapy. Enoblituzumab (MGA271) is a humanized IgG1 mAb that binds to B7-H3 with high affinity (15). The antibody has been engineered to display enhanced binding to the activating FcγR (CD16A), as well as to the low-affinity allele of CD16A, CD16A-158F. As most individuals carry the low-affinity allele of CD16A, the improved binding of the Fc-optimized enoblituzumab should facilitate antibody-dependent cell-mediated cytotoxicity (ADCC) in all patients, except for the small cohort (∼15%) who are homozygous for the high-affinity allele of CD16A. Moreover, enoblituzumab has been designed to exhibit reduced binding to the low-affinity inhibitory FcγR receptor, CD32B (15).

Most published data support an inhibitory role for B7-H3 on T-cell and NK cell function (16–22). Thus, in addition to facilitating ADCC by binding to B7-H3, enoblituzumab may block this immune checkpoint and further promote immune-mediated tumor eradication.

Enoblituzumab was tested in 146 adults with B7-H3–expressing tumors in a phase I dose escalation study evaluating safety and tolerability (23). Enoblituzumab was well tolerated, with toxicities manageable by standard medical therapy, at doses up to 15 mg/kg administered weekly by intravenous infusion. No dose-limiting toxicities (DLT) were observed at any dose level, and the majority of adverse events (AE) were mild (grade 1 or 2). Moreover, some patients experienced disease stabilization (>12 weeks) and tumor shrinkage (2%–69%) across disease histologies.

We report here the results of the first phase I trial utilizing enoblituzumab in children and young adults with B7-H3–expressing relapsed or refractory solid tumors to determine the maximum tolerated dose (MTD) or maximum administered dose (MAD), toxicity profile, pharmacokinetics (PK), pharmacodynamics, and immunogenicity of this antibody.

## Materials and Methods

### Patient population

Criteria for eligibility included children and young adults, ages 1 to 35 years, with relapsed or refractory solid tumors of any histology with measurable disease per RECIST 1.1 criteria and documented by CT and/or MRI, or patients with neuroblastoma and nonmeasurable disease defined as evaluable only by meta-iodobenzylguanidine scintigraphy and/or bone marrow histology. Patients were required to have a Karnofsky or Lansky performance score of ≥70% and a life expectancy of >12 weeks. Detailed inclusion and exclusion criteria from the clinical protocol are included in Supplementary Data File S2. Written informed consent was obtained from patients (ages ≥18 years) or their parents/legal guardians for patients <18 years. The study was conducted with the approval of the Institutional Review Board at each participating site.

### Tumor analysis

Tumor samples (fresh or formalin-fixed, paraffin-embedded) were analyzed for B7-H3 expression by IHC in a central laboratory (MacroGenics core facility). The percentage of cancer cells and tumor vascular staining 1+ (weak), 2+ (moderate), or 3+ (strong) was determined. Patients’ tumors had to demonstrate B7-H3 expression at 2+ or greater levels on the membranous surface of at least 10% of tumor cells or ≥25% of tumor vasculature to be considered “B7-H3+” and eligible for treatment in this trial.

### Study design

This multicenter, open-label, single-arm phase I trial was carried out in six U.S. academic hospitals. The primary objective of the study was to characterize the safety, tolerability, DLTs, and MTD (or the MAD if no MTD is defined) of enoblituzumab when given weekly to children and young adults with B7-H3–expressing relapsed or refractory solid tumors of any histology. Secondary objectives included characterization of the PK and immunogenicity of enoblituzumab in pediatric patients when administered intravenously on a weekly schedule. In addition, pharmacodynamics and preliminary antitumor activity of enoblituzumab were examined using both conventional RECIST 1.1 criteria and immune-related RECIST (irRECIST; refs. 24, 25). The study consisted of a dose escalation phase to determine the MTD or MAD, followed by a cohort expansion phase to further define the safety and assess the preliminary antitumor activity of enoblituzumab. The MTD was defined as the highest dose level tested at which <33% of patients experienced a drug-related DLT during the DLT evaluation period, defined as the time from administration of the first dose until 7 days after the administration of the fourth dose. Enoblituzumab was administered over 120 minutes after patients were premedicated with diphenhydramine, acetaminophen, and hydrocortisone. If no infusion reaction was observed with the first dose, then hydrocortisone was omitted as a premedication for subsequent doses. The initial dose of enoblituzumab was 10 mg/kg, which is lower than the adult MTD (15 mg/kg). Starting at a lower dose was felt to be prudent as the antibody had not previously been administered to pediatric patients. If <33% of patients experienced a DLT at this dose level, then the dose would be escalated to the maximum protocol-specified dose of 15 mg/kg. If <33% of patients experienced a DLT at the 15 mg/kg dose during the dose escalation phase of the trial, then that dose level was designated as the MAD. Definitions of DLTs are provided in Supplementary Data File S1.

The cohort expansion phase involved weekly infusions of enoblituzumab at the MTD or MAD to patients who were assigned to one of five cohorts based on disease type: (i) neuroblastoma (measurable disease), (ii) neuroblastoma (nonmeasurable disease), (iii) rhabdomyosarcoma, (iv) osteosarcoma, and (v) Ewing sarcoma, Wilms tumor, desmoplastic small round cell tumor, or malignant solid tumors of any other histology that test positive for B7-H3. The intent was to enroll 10 patients per cohort.

Safety assessments were performed on all patients who received at least one dose of enoblituzumab and were based on the evaluation of treatment-emergent AEs, using Common Terminology Criteria for Adverse Events version 4.03. All patients who received at least one dose of enoblituzumab and had at least one disease assessment following any treatment were evaluated for response. Disease assessment occurred after the eighth enoblituzumab infusion and every 8 weeks thereafter. Disease assessment in patients with neuroblastoma employed the neuroblastoma overall response criteria, which integrates disease measurable at all sites, namely bone marrow, ^123^I-metaiodobenzylguanidine avid lesions, catecholamines, and CT/MRI lesions (26). Patients who achieved an objective response status of stable disease, partial response (PR), or complete response (CR) were eligible to receive subsequent doses of enoblituzumab, assuming they remained clinically stable and did not experience unacceptable toxicity that necessitated permanent discontinuation of the drug.

### Pharmacokinetic measurements, pharmacodynamics, and immunogenicity studies

Serum concentrations of enoblituzumab were monitored using a quantitative sandwich ELISA (MacroGenics core facility). Serum cytokine levels were measured by a qualified Luminex multiplex immunoassay (MacroGenics core facility). The generation of antidrug antibodies (ADA) directed against enoblituzumab was assayed using ELISA (MacroGenics core facility).

### Population pharmacokinetic modeling

Model building and evaluation were conducted using NONMEM version 7 level 4 (ICON Development Solutions), and standard model building approaches were followed to define the structure, intersubject variability, and covariate dependence of the model. Modeling was performed on a Dell Inspiron E6410 i7 laptop running the current gfortran compiler. R, version 3.2.3 or higher, was used for statistical modeling and graphics in combination with Xpose4. Wings for NONMEM was used as an interface for model fitting and bootstrap simulations. Five hundred bootstrap runs were performed using the Nonmen Batch System routine in Wings for NONMEM using the parameters from the final model to provide 95% confidence intervals for the parameters. Covariate factors that had 95% confidence intervals that included null were removed, and the bootstrap was rerun. Additionally, a visual predictive check was conducted to compare the distribution of simulated observations from the final model to those obtained from the original data. In this visual check, the concentration–time profiles were simulated using the final model parameters. In addition to the plots of interindividual variability, the normalized prediction distribution error was examined and plotted against covariates.

### Statistical analyses

This phase I study was primarily observational, and thus, most of the reported results are descriptive. Response rates were to be determined using both conventional RECIST 1.1 and irRECIST, and neuroblastoma overall response criteria were to be determined using conventional RECIST 1.1 and irRECIST to assess CT/MRI evaluable lesions. Response rates using irRECIST were not determined as no CRs or PRs were observed. For primary analyses of serum cytokine levels, a linear mixed model for repeated measures over time was utilized for each cytokine. This procedure prevents listwise deletion due to randomly missing data. Dunnett’s multiple comparisons test was performed *post hoc* to analyze differences between baseline and postinfusion serum cytokine levels. *P* values < 0.05 were considered significant.

### Data availability

Raw data for this study were generated by MacroGenics and are no longer available. Derived data supporting the findings of this study are available from the corresponding author upon request.

## Results

### IHC screening for B7-H3 expression

There were 101 patients whose tumors were submitted for B7-H3 IHC staining. The results of this testing are shown in Table 1. Of the 101 submitted tumor samples, 8 were not evaluable because of technical issues. Of the 93 patients who had tumors with technically successful IHC staining, 89 (96%) tested positive for B7-H3 expression. Only 4 of the 89 tumor specimens with successful IHC staining tested negative for B7-H3 expression; all four of these B7-H3–negative samples were from the 15 patients with nonmeasurable neuroblastoma. Hence, except for these four patients with nonmeasurable neuroblastoma, every other patient with IHC-evaluable specimens submitted for screening had tumors that expressed B7-H3, including all cases of measurable neuroblastoma, osteosarcoma, rhabdomyosarcoma, DRSCT, and Ewing sarcoma and all 13 patients with other, less common solid tumors.

**Table 1:** IHC staining for B7-H3 expression in screened patients

Tumor type	Total	Positive	Negative	Rate of positivity (%)
Neuroblastoma (NM)^a^	15	11	4	73
Neuroblastoma (M)^a^	13	13	0	100
Osteosarcoma	21	21	0	100
Rhabdomyosarcoma	14	14	0	100
Desmoplastic small round cell tumor	8	8	0	100
Ewing sarcoma	9	9	0	100
Other^b^	13	13	0	100
Study totals	93	89	4	96

a M, measurable disease; NM, nonmeasurable disease.

b These tumors were alveolar soft part sarcoma (*n* = 3), synovial sarcoma (*n* = 2), and other sarcoma (*n* = 3) and one patient each with Wilms tumor, hepatocellular carcinoma, germ cell tumor, Hodgkin lymphoma, melanoma, and undifferentiated small round blue cell tumor.

More detailed information about IHC staining for the patients who received enoblituzumab is presented in Supplementary Tables S1 and S2. When including a positivity cutoff of 1+ B7-H3 expression, 14 out of 24 treated patients (58%) demonstrated B7-H3 detectable on all of their tumor cells, 17 out of 24 (71%) demonstrated B7-H3 on at least 95% of their tumor cells, 21 out of 24 (88%) demonstrated B7-H3 on at least 90% of their tumor cells, and all patients demonstrated B7-H3 detectable on at least 70% of their tumor cells. Similarly, tumor vasculature stained positive for B7-H3 in all 23 samples tested (no blood vessels could be identified in one sample). In 14 of 23 samples, moderate or strong B7-H3 expression was documented in ≥40% of tumor vasculature. These results indicate the widespread expression of B7-H3 on the majority of evaluable cancer cells for nearly all pediatric patients with solid tumors treated on this trial.

### Patient characteristics

A total of 25 patients were enrolled in the study, and 24 received enoblituzumab. Disease cohort accrual goals were not met because of early trial closure by the study sponsor. The median age of treated patients was 14.8 years (range 6–24 years). Most patients were male (*n* = 18), and 18 patients were White. The most common diagnoses were neuroblastoma (*n* = 8) and osteosarcoma (*n* = 6). A summary of demographics and baseline patient characteristics is presented in Table 2. Most patients (67%) had metastatic disease at diagnosis, and all patients had developed metastatic disease prior to study entry (Table 3).

**Table 2:** Patient characteristics (*n* = 24; median, range)

Age, years	14.8 (6–24)	
Sex	​	
Male	18 (75%)	
Female	6 (25%)	
Weight (kg)	58.0 (19.2–181.6)	
Height (cm)	155.3 (120.2–188.5)	
Race	​	
White	18 (75%)	
Asian	0	
American Indian or Alaska Native	1 (4%)	
Black or African American	3 (13%)	
Latino	1 (4%)	
Unknown	1 (4%)	
Tumor type (for 24 patients receiving enoblituzumab)	Dose escalation	Cohort expansion
Neuroblastoma (measurable)	0	1
Neuroblastoma (nonmeasurable)	4	3
Osteosarcoma	0	6
Ewing sarcoma, Wilms tumor, or desmoplastic small round cell tumors	0	6
Rhabdomyosarcoma	0	2
Hepatocellular carcinoma	1	0
Undifferentiated sarcoma	1	0
Lansky/Karnofsky performance scale score [*n* (%)]	​	​
60	1 (4%)	
70	4 (17%)	
80	2 (8%)	
90	12 (50%)	
100	5 (21%)	

**Table 3:** Patient disease status at diagnosis and study entry

Patient	Diagnosis	Disease status at diagnosis	Site(s) of metastatic disease at study entry
1	HCC	Metastatic	Lungs
2	NBNM	Metastatic	Bone, brain
3	NBNM	Metastatic	Bone
4	NBNM	Metastatic	Bone, lymph nodes, pleura
5	NBNM	Metastatic	Bone
6	Liver sarcoma	Localized	Liver, lungs
7	NBNM	Metastatic	Bone
8	NBNM	Metastatic	Bone
9	NB	Metastatic	Bone, soft tissue
10	NBNM	Metastatic	Bone
11	RMS	Localized	Lymph node, soft tissue
12	RMS	Localized	Lungs
13	OS	Metastatic	Lungs, lymph nodes
14	OS	Metastatic	Lungs
15	OS	Metastatic	Lungs
16	OS	Metastatic	Lungs
17	OS	Localized	Lungs
18	OS	Localized	Lungs, bone
19	DSRCT	Metastatic	Brain
20	Melanoma	Local	Skin
21	USRBCT	Local	Lungs
22	Synovial sarcoma	Local	Lungs
23	DSRCT	Metastatic	Liver, lungs, lymph nodes
24	Ewing	Metastatic	Lungs

Abbreviations: DSRCT, desmoplastic small round cell tumor; HCC, hepatocellular carcinoma; NB, neuroblastoma; NBNM, neuroblastoma with nonmeasurable disease; OS, osteosarcoma; RMS, rhabdomyosarcoma; USRBCT, undifferentiated small round blue cell tumor.

### Dose escalation and summary of drug exposure

In the dose escalation phase of this trial, three patients received the 10 mg/kg dose of enoblituzumab, and three patients received the 15 mg/kg dose. No DLTs were observed at the 10 or 15 mg/kg dose levels, so all subsequent patients were treated at the MAD of 15 mg/kg. The median number of doses administered was 6.5 (range 1–24). Administration of enoblituzumab was interrupted at some point in approximately one third of patients because of infusion-related toxicities. However, the infusion was resumed in almost all cases, and the dose intensity, defined as the percentage of total dose administered divided by the total dose intended for the whole treatment period, was 99%. Guidelines for the management of infusion-related reactions are provided in Supplementary Data File S3. Details of the enoblituzumab doses administered are provided in Table 4.

**Table 4:** Enoblituzumab exposure for the 24 treated patients

​	Total (*n* = 24)
Number of patients dosed, *n* (%)	24 (100)
Any infusion interruptions, *n* (%)	​
Yes	8 (33.3)
No	16 (66.7)
Number of doses received	​
Mean ± SD	7.3 ± 5.45
Median (range)	6.5 (1.0–24.0)
Number of doses/patient	Number of patients (%)
1	1 (4.2)
2	1 (4.2)
3	2 (8.3)
4	3 (12.5)
5	2 (8.3)
6	3 (12.5)
7	1 (4.2)
8	9 (37.5)
≥12	2 (8.3)
Dosing duration (days)	​
Mean ± SD	51.0 ± 39.0
Median (range)	49.5 (10.0–172.0)
Total dose administered (mg)	​
Mean ± SD`	5,615.5 ± 5,003.4
Median (range)	3,332.3 (1,000.0–21,616.0)
Total dose intended (mg)	​
Mean ± SD	5,673.6 ± 5,009.4
Median (range)	3,332.3 (1,000.0–21,616.0)
Dose intensity	​
Mean ± SD	99.1 ± 2.8
Median (range)	100.0 (89.2–100.0)

### Toxicities

All patients experienced at least one AE (Table 5). A total of 21 patients (87.5%) experienced at least one treatment-related AE (TRAE). The most common TRAE was infusion reaction, including fever, chills, hypotension, and rash, which occurred in nine patients (37.5%). A total of 14 patients (58.3%) experienced grade ≤2 TRAEs, and seven patients (29.2%) experienced grade 3 TRAEs. Grade 3 TRAEs included anaphylactoid reaction (two patients), cytokine release syndrome (two patients), hypophosphatemia, infusion-related reaction, low neutrophil count, pericardial effusion, and urinary tract obstruction (one patient each). There were no grade 4 or 5 TRAEs observed in this trial. All grade 3 TRAEs and grade 1 to 2 TRAEs occurring in three or more patients reported during the study period are summarized in Table 6. Twelve patients (50%) experienced 13 AEs that met protocol-defined criteria for AEs of special interest. These included anaphylactoid reactions (two patients), cytokine release syndrome (two patients), and infusion-related reactions (nine patients).

**Table 5:** AEs (regardless of causality) by system/organ class

System/organ class	Total (*N* = 24), *n* (%)
Number of patients with at least one event	24 (100)
General disorders and administration site conditions	​
Pyrexia	9 (37.5)
Fatigue	6 (25.0)
Noncardiac chest pain	5 (20.8)
Musculoskeletal and connective tissue disorders	​
Pain in extremity	6 (25.0)
Arthralgia	3 (12.5)
Immune system disorders	​
Anaphylactoid reaction	2 (8.3)
Cytokine release syndrome	2 (8.3)
Urinary disorders	​
Urinary tract obstruction	1 (4.2)
Gastrointestinal disorders	​
Vomiting	8 (33.3)
Nausea	6 (25.0)
Constipation	3 (12.5)
Injury, poisoning, and procedural complications	​
Infusion-related reaction	9 (37.5)
Metabolism and nutrition disorders	​
Decreased appetite	5 (20.8)
Hypoalbuminemia	5 (20.8)
Hyperglycemia	4 (16.7)
Respiratory, thoracic, and mediastinal disorders	​
Cough	4 (16.7)
Epistaxis	3 (12.5)
Nasal congestion	3 (12.5)
Pleural effusion	1 (4.2)
Laboratory investigations	​
Lymphocyte count decreased	5 (20.8)
Aspartate aminotransferase increased	4 (16.7)
Blood creatinine increased	4 (16.7)
White blood cell count decreased	3 (12.5)
Hemoglobin decreased	6 (25.0)
Nervous system disorders	​
Headache	8 (33.3)
Mental status changes	1 (4.2)
Skin and subcutaneous tissue disorders	​
Pruritus	4 (16.7)
Cardiac disorders	​
Sinus tachycardia	3 (12.5)
Pericardial effusion	1 (4.2)
Infections	​
Viral infection	1 (4.2)

**Table 6:** TRAEs (*n* = 24)

AE^a^	Grades 1/2^b^	Grade 3^b^
Headache	4 (16.7%)	0
White blood cell count decreased	3 (12.5%)	0
Nausea	3 (12.5%)	0
Vomiting	4 (16.7%)	0
Pyrexia	5 (20.8%)	0
Infusion-related reaction	8 (33.3%)	1 (4.2%)
Anaphylactoid reaction	0	2 (8.3%)
Cytokine release syndrome	0	2 (8.3%)
Hypophosphatemia	0	1 (4.2%)
Neutrophil count decreased	0	1 (4.2%)
Pericardial effusion	0	1 (4.2%)
Urinary tract obstruction	0	1 (4.2%)

There were no grade 4 or 5 TRAEs reported in this study.

a AEs that were definitely, probably, or possibly related to study treatment.

b Grade 1/2 AEs occur in ≥10% of patients, and all grade 3 events are listed.

### Pharmacokinetics

Pharmacokinetic analysis of enoblituzumab revealed that the concentrations in plasma after a 2-hour infusion were described well by a linear, two-compartment model. The model was parameterized in terms of clearance (CL), volume of distribution of the central and tissue compartments (V_1_, V_2_), and clearance of distribution. Between-subject variability was applied to both CL and V_1_. Covariates of dose, age, weight, sex, lean weight, race, and performance status were evaluated for importance in describing CL and V_1_, but none showed significance. However, this could be due to the relatively small number of patients enrolled in the study. The PK parameters for the model are provided in Supplementary Table S3, with parameters from the fitted data and the 500-iteration bootstrap of the model compared. The parameters are in close agreement despite the inclusion of the two outlier subjects in the bootstrap, suggesting a robust PK model.

No dose dependency of the clearance or distribution was noted, and BSA (or weight) seemed to significantly affect only the distribution of the drug in the plasma in the available data. The final PK model included a proportional effect of BSA on the central distribution volume. The drug was dosed on a mg/kg basis, and in fact, the PK model fit well with weight as a covariate for distribution volume. BSA was used in the final model due to an improved fit based on the objective function, but the difference was minimal.

**Figure 1:**
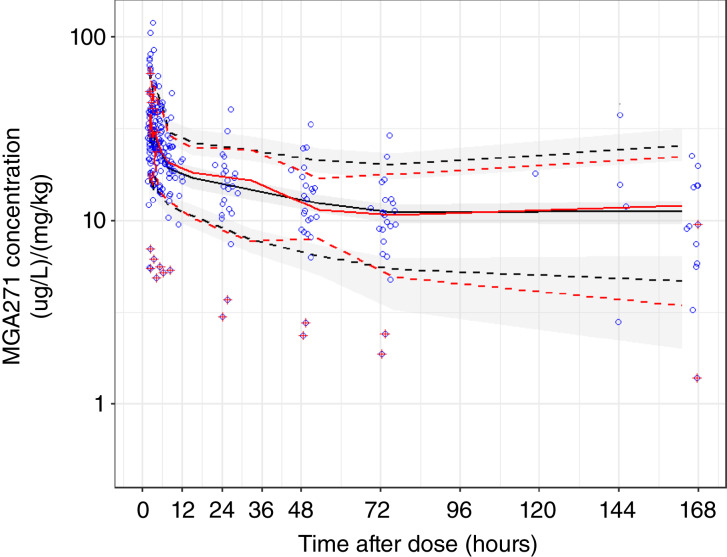
Normalized prediction distribution errors and visual prediction plots present the simulated values for the median concentration (black solid line) and 90th percentile concentration (black dashed lines), with the simulated mean and 90th percentile intervals indicated by the gray shaded ribbons. The median and 90th percentile values for the observed concentrations are the red solid and dashed lines. The two outlier subjects are shown as red crosses.

Normalized prediction distribution errors and visual prediction plots suggested the acceptability of the final model (Fig. 1). Most observed values fall well within the confidence interval of the simulated data, suggesting an appropriate PK model for the drug. The geometric mean and range of the elimination half-life of the drug were 67 (7–900) days.

### ADA testing

Testing for antibodies generated against enoblituzumab was performed at weeks 1, 3, 7, and 14 and at the end of the treatment. ADAs were only detected in one patient at week 1, but not at later time points (weeks 3, 7, and 14 and the end of the treatment) when followed longitudinally.

### Cytokine levels

**Figure 2:**
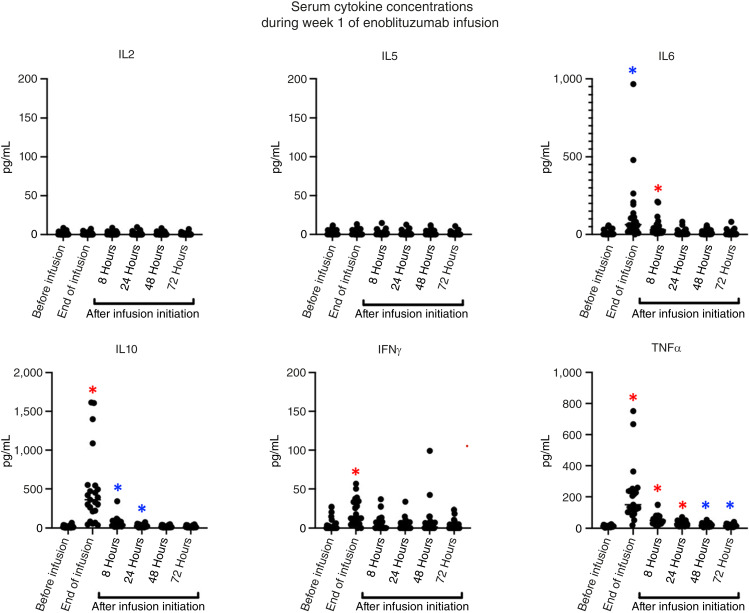
Serum cytokine concentrations during week 1 of enoblituzumab infusion. Serum cytokine concentrations were tested in all subjects at the end of the infusion and at 8, 24, 48, and 72 hours after starting the infusion. Cytokine concentration compared with preinfusion cytokine concentration is different at a significance level of *, *P* < 0.05 or *, *P* < 0.001.

Cytokine levels of IL2, IL5, IL6, IL10, IFNγ, and TNFα were measured by Luminex multiplex immunoassay at baseline (before infusion), at the end of the enoblituzumab infusion, and at 8, 24, 48, and 72 hours after initiation of infusion for the first (week 1) enoblituzumab dose (Fig. 2). Levels of IL6 were found to be significantly elevated at the end of infusion and 8 hours after infusion but returned to baseline by 24 hours after infusion. Levels of IL10 were markedly elevated at the end of infusion and remained elevated for 24 hours after infusion. Levels of TNFα were markedly elevated at the end of infusion and remained elevated at all time points evaluated. No changes were seen in the levels of IL2 or IL5. There was no association observed between cytokine levels and cytokine release syndrome or other infusion-related toxicities (data not shown).

### Tumor response

There were 21 patients who were evaluable for tumor response. No CRs or PRs were observed. The best response was stable disease, documented in two patients.

## Discussion

In this study, we demonstrated the tolerability of the MAD of enoblituzumab, 15 mg/kg, which was the dose utilized for adult patients in phase II trials for prostate and head and neck cancers (NCT02923180, NCT04634825). The main TRAEs, which occurred during the infusion of the drug, were typically transient, and the full dose could be administered in nearly all instances.

B7-H3 seems to be a good target for immunotherapeutic strategies directed against both adult and pediatric cancers. It is highly expressed on a wide variety of tumor histologies, whereas expression on normal tissues is reportedly low (5), thereby minimizing the risk of on-target, off-tumor toxicities. We identified B7-H3 expression in 96% of cases screened when excluding the 8 of 101 cases in which IHC testing was either not performed or was technically unsuccessful. More detailed IHC data are presented for the 24 patients treated with enoblituzumab. All samples demonstrated B7-H3 expression in both cancer cells and tumor vasculature, with 21 of 24 samples showing moderate or strong staining in ≥40% of cancer cells, and all samples demonstrated detectable B7-H3 expression in ≥70% of cancer cells. Similarly, Kendersky reported high expression of B7-H3 on patient-derived xenografts generated from a variety of pediatric cancers and low B7-H3 expression on normal tissues (27). This high degree of tumor-associated antigen expression, across such a wide array of disease histologies, bestows a unique status on B7-H3 as an immunotherapeutic target for pediatric solid tumors.

High B7-H3 expression has been associated with worse clinical outcomes for some cancers, including non–small cell lung cancer, breast cancer, colorectal cancer, prostate cancer, and osteosarcoma (8, 9, 12, 13). Expression of B7-H3 has been shown to promote tumor migration and invasiveness (8, 28–30). There is also evidence suggesting that B7-H3 serves as a checkpoint molecule inhibiting both T-cell and NK cell function (18, 20–22). In osteosarcoma, the level of B7-H3 expression has been shown to inversely correlate with the number of tumor-infiltrating CD8^+^ T cells (8). In addition, blocking B7-H3 expression with 5B14, an anti–B7-H3 IgM mAb, led to enhanced NK cell–mediated killing of neuroblastoma cells freshly isolated from the bone marrow of newly diagnosed stage M patients (1).

Enoblituzumab is a humanized IgG1 mAb that has been engineered for optimized binding through its FcγR. Hence, the antibody may exert an antitumor effect by facilitating ADCC or by blocking B7-H3–mediated immune evasion. This antibody demonstrated potent ADCC against a variety of tumor cell lines *in vitro* and showed significant antitumor activity in a murine xenograft model (15). However, there were no objective responses observed in this trial, in which patients with B7-H3–positive relapsed or refractory solid tumors were treated with weekly infusions of enoblituzumab. There are several possible explanations for this. mAbs that rely on ADCC for their therapeutic action may not be effective in pediatric patients with solid tumors harboring gross residual disease. For example, one of the most widely used mAbs for the treatment of neuroblastoma, dinutuximab, primarily showed single-agent therapeutic benefit when administered to patients with minimal residual disease though a higher objective response rate was seen in patients with neuroblastoma with measurable disease utilizing the humanized anti-GD2 mAb, 3F8 (31–33). The inability of many mAbs to mediate significant antitumor responses in patients with bulky disease may relate to poor antibody penetration within the tumor, as well as the highly immunosuppressive tumor microenvironment, which dampens antibody-mediated cellular responses. The other potential mechanism of action for enoblituzumab, checkpoint blockade, has not proven to be an effective strategy against the majority of pediatric cancers. This is thought, at least in part, to be a consequence of the low mutational burden found in most pediatric malignancies (34). The lower mutational burden results in fewer neoantigens that can generate an adaptive immune response.

Despite these obstacles, enoblituzumab remains a potentially attractive drug to use against pediatric cancers, given the uniformly high levels of B7-H3 expression across a wide range of tumor histologies. A number of strategies could be considered to improve the efficacy of this agent. Our study was designed to evaluate the safety and preliminary antitumor activity of enoblituzumab administered at the recommended adult dose of 15 mg/kg. The MTD of this mAb was not established in children, and it is possible that utilizing higher doses of enoblituzumab may have resulted in greater antitumor activity. Combining enoblituzumab with low-dose cytotoxic chemotherapy is another strategy that could be explored. The administration of chemotherapy with an antitumor mAb has yielded impressive results in the treatment of relapsed/refractory neuroblastoma (35, 36). The combined use of an anti-GD2 mAb and chemotherapy has also been tested in patients with newly diagnosed high-risk neuroblastoma with encouraging results (37).

Another strategy to improve efficacy could be to combine enoblituzumab with checkpoint blockade. Recent data indicate that the combination of ADCC-inducing mAbs with anti-PD1 treatment can augment *in vivo* ADCC, in both preclinical and clinical testing. A phase I trial of enoblituzumab, administered in combination with pembrolizumab, in adults with various malignancies showed acceptable tolerance and promising antitumor activity (38).

Although enoblituzumab has demonstrated significant cytotoxicity *in vitro* and in preclinical murine models, the lack of clinical activity seen in this trial may have, at least in part, been secondary to our patient population harboring relatively high tumor burdens and bearing immune systems compromised by multiple prior therapies. It is conceivable that enoblituzumab would be more effective if used in a different clinical setting, such as maintenance therapy for patients who are at high risk of relapse following completion of standard therapy. The presence of metastatic disease at diagnosis portends a poor prognosis for most patients with sarcoma, and this might be an ideal population to test an immunotherapy maintenance strategy. Indeed, this was the approach that led to FDA approval of dinutuximab for the treatment of high-risk neuroblastoma (32). In an adult trial, enoblituzumab was administered weekly for 6 weeks to men with high-risk prostate cancer prior to radical prostatectomy with the intention of eliminating micrometastatic disease (39). The declines in Gleason-grade scores before prostatectomy following treatment with enoblituzumab, as well as the prostate-specific antigen recurrence rate 1 year after prostatectomy, suggested a benefit from utilizing adjuvant immunotherapy in these patients. However, additional preclinical studies would likely be warranted prior to testing enoblituzumab as a maintenance therapy for high-risk pediatric sarcoma patients.

Finally, enoblituzumab or other B7-H3–specific mAbs could be conjugated to a radionuclide or drug/toxin in an attempt to improve efficacy. For example, 8H9, a murine IgG1 anti–B7-H3 mAb, has been conjugated to I-131 and proved efficacious, as one component of a multiagent regimen, in the treatment of patients with neuroblastoma who experienced a central nervous system relapse (40). For these children, the radioimmunoconjugate was injected directly into the central nervous system through an Ommaya reservoir and localized to sites of leptomeningeal disease. A number of B7-H3 antibody–drug conjugates (ADC) are also under development. Vobramitamab duocarmizine, a B7-H3 ADC, demonstrated potent antitumor activity in a number of preclinical patient-derived xenograft models and showed a favorable PK and safety profile in cynomolgus monkeys (41). This ADC also mediated significant cytotoxicity against neuroblastoma cells in monolayer and multicellular tumor spheroid models, as well as prolonged survival of mice orthotopically implanted with B7-H3+ neuroblastoma cell lines (42).

In addition to anti–B7-H3 mAbs, a number of chimeric antigen receptor T-cell constructs directed against B7-H3 have been developed, and some are currently undergoing clinical testing (43–45). A comprehensive review of B7-H3 targeting strategies for pediatric cancer has been published by Rasic (46).

In summary, we found robust expression of B7-H3 on the vast majority of tumors screened for this phase I trial. An enoblituzumab dose of 15 mg/kg administered once a week was safe and had a PK profile similar to what has been reported for adult patients. Enoblituzumab induced systemic immune activation as evidenced by infusion-related reactions, cytokine release syndrome in two patients, and inflammatory cytokine induction. Our data support B7-H3 as an excellent target for immunotherapy strategies directed against pediatric solid tumors and suggest that enoblituzumab may warrant further testing in the setting of minimal residual disease and/or in combination with other anticancer agents.

## Supplementary Material

Supplementary Data File 1 DLT Definitions

Supplementary Data File 2 Inclusion/Exclusion criteria

Supplementary Data File 3 Guidelines for the Management of Infusion-Related Reactions

Supplementary Table 1 Supplementary Table 1. B7-H3 Expression Patterns for Cancer Cells (CC) and Tumor Vasculature (VAS)

Supplementary Table 2 Supplementary Table 2. Average B7-H3 Expression Patterns for Treated Patients by Diagnosis

Supplementary Table 3 Supplementary Table 3. Parameter Estimates for Final Pharmacokinetic Model

Supplementary Table 4 Supplementary Table 4. Representativeness of Study Participants
