# Supplementary material for: A Phase I, Open-Label, Dose Escalation Study of Enoblituzumab in Children and Young Adults with B7-H3–Expressing Relapsed or Refractory Solid Tumors
Source: Cancer Res Commun. 2025 Sep 10;5(9):1574–83. doi: 10.1158/2767-9764.CRC-25-0293 (PMC12421222; doi:10.1158/2767-9764.CRC-25-0293)
Supplement: Supplementary Data File 1 — DLT Definitions [file crc-25-0293_supplementary_data_file_1_suppsd1.pdf]

Definition of Dose Limiting Toxicities

In patients who experience an AE that might meet the criteria for a DLT, administration of additional MGA271 should be held pending management and/or resolution of the event, and the assessment of causal attribution to study drug. Whether a DLT is considered related to study drug will be determined by the Investigator. An adverse event must be considered possibly, probably, or definitely related to study drug in order to be classified as a DLT. No patient dose reductions are allowed on the study except in cases where patients are receiving a dose level that is subsequently deemed to have exceeded the MTD. Criteria for the subsequent continuation of therapy are outlined below (Section 5.1.6).

For the purpose of guiding decisions regarding dose escalation, DLTs will be defined based on drug-related AEs (or laboratory abnormalities) that occur during the DLT evaluation period defined as the period from administration of the first dose of MGA271 (Day 1) until 7 days after the administration of the fourth dose of MGA271(Day 29). During the DLT evaluation period, patients will need to have received at least 4 doses of MGA271, or experienced a DLT, to be considered evaluable. The severity of adverse events will be graded according to the National Cancer Institute Common Terminology Criteria for Adverse Events v 4.03 (CTCAE v 4.03).

Dose limiting toxicities are defined separately for hematologic and non-hematologic events as outlined in the sections below.

Hematologic Dose Limiting Toxicity

Hematologic DLT will be defined as follows:

- Grade 4 neutropenia lasting > 5 days
- 2 Grade 3 febrile neutropenia lasting > 48 hours or any 2 Grade 3 febrile neutropenia associated with hemodynamic compromise or objective evidence of infection
- Grade 4 thrombocytopenia, irrespective of duration
- Grade 3 thrombocytopenia associated with clinically significant bleeding
- 2 Grade 3 hemolysis

The following events will be specifically excluded from the definition of hematologic DLT:

- 2 Grade 3 lymphopenia
- Grade 3 anemia that is not associated with other clinically significant complications

Non-Hematologic Dose Limiting Toxicity

Non-hematologic DLT will be defined as any 2 Grade 3 non-hematologic event with the following exceptions:

- Grade 3 electrolyte abnormality that lasts < 72 hours, is not otherwise associated with clinical complications, and responds to medical intervention
- Grade 3 fever that lasts < 72 hours and is not associated with hemodynamic compromise
- Grade 3 nausea or vomiting that lasts < 72 hours and responds to medical intervention
- Grade 3 amylase and/or lipase elevation that is not associated with either clinical or radiographic evidence suggestive of pancreatitis
- Grade 3 gastrointestinal AEs of diarrhea, constipation, abdominal pain, cramping, dyspepsia or dysphagia that resolves to Grade 1 within 14 days with medical therapy
- Grade 3 fatigue that lasts < 7 days
- Grade 3 infusion-related reaction or cytokine release syndrome that lasts < 12 hours and responds to medical intervention.
- Grade 3 or 4 endocrinopathy that is adequately controlled with hormone supplementation
- Grade 3 skin toxicity that resolves to Grade 2 within 14 days of initiation of oral corticosteroids
- Grade 3 inflammatory reaction (e.g., with associated pain, swelling) attributed to a local antitumor response (e.g., inflammatory reaction at sites of metastatic disease, lymph nodes, etc.) that resolves to Grade 2 within 7 days

Note: The following Grade 2 or greater non-hematologic AE may also be considered as DLT:

- Grade 2 AEs that are prolonged inordinately, based upon the medical judgment of the Investigator, and/or lead to permanent discontinuation of MGA271 due to patient intolerance
- Any Grade 2 eye pain or reduction in visual acuity that does not respond to topical therapy and does not improve to Grade 1 within 14 days of the

initiation of topical therapy, or that requires systemic treatment

Hepatic Non-Hematologic Dose Limiting Toxicity

- Any elevation of one or more transaminases > 8 x the institutional upper limit of normal (ULN) irrespective of duration
- Any Grade 3 elevation of one or more transaminases > 5.0 - 8.0 x the ULN that does not resolve to Grade 2 (i.e., >2.5 - 5.0 x ULN) within 7 days and Grade 1 (i.e., >ULN - 2.5 x ULN) within 14 days. In addition, steroids must be tapered to10 mg of prednisone or equivalent per day, by Day 14
- A Grade 3 elevation of total bilirubin that is > 5 x the ULN irrespective of duration
- Any Grade 3 elevation of total bilirubin >3.0 - 5.0 x ULN that does not resolve to Grade 2 (i.e., >1.5 - 3.0 x ULN) within 7 days and Grade 1 (i.e., >ULN - 1.5 x ULN) within 14 days. In addition, steroids must be tapered to 10 mg of prednisone or equivalent per day, by Day 14
- Any event meeting the criteria for Hy's law as follows (all 3 features):
    - Aspartate aminotransferase (AST) and/or alanine aminotransferase (ALT) > 3 x ULN
    - Concurrent elevation of total bilirubin > 2 x ULN without initial evidence of cholestasis
    - No alternative etiology can be identified

An infusion delay of > 14 days due to drug-related toxicity during the DLT observation period in the Dose Escalation Phase will constitute a DLT. The study drug will be discontinued and the patient should complete the end of study visit.
