# Supplementary material for: A Phase I, Open-Label, Dose Escalation Study of Enoblituzumab in Children and Young Adults with B7-H3–Expressing Relapsed or Refractory Solid Tumors
Source: Cancer Res Commun. 2025 Sep 10;5(9):1574–83. doi: 10.1158/2767-9764.CRC-25-0293 (PMC12421222; doi:10.1158/2767-9764.CRC-25-0293)
Supplement: Supplementary Data File 2 — Inclusion/Exclusion criteria [file crc-25-0293_supplementary_data_file_2_suppsd2.pdf]

Inclusion and Exclusion Criteria:

Inclusion Criteria:

General:

1. 1 to 21 years of age at diagnosis of primary tumor (treatment up to the age of 30 years old)
2. Ability of patient or parent/guardian of patient to provide informed consent and documentation of informed consent prior to initiation of any study-related tests or procedures that are not part of standard care. Parent/guardians must also be willing and able to comply with study procedures, including the acquisition of specified research specimens. Where appropriate, based on the age of the patient, assent should also be sought and documented for patients if informed consent is provided by the parent or guardian, per 21CFR50.55
3. Relapsed or refractory malignant solid tumors of any histology for which no standard curative therapy is available (escalation phase); Expansion phase will be limited to histologically proven:

    a) Neuroblastoma

        i. Measurable disease- defined as disease measurable by standard radiographic criteria
        ii. Non-measurable disease- defined as disease evaluable only by MIBG scintigraphy and/or bone marrow histology

    b) Rhabdomyosarcoma

    c) Osteosarcoma

    d) Ewing's sarcoma/Primitive neuroectodermal tumor

    e) Wilms tumor

    f) Desmoplastic small round cell tumor

4. Patients must have malignant solid tumors that demonstrate B7-H3 expression at 2+ or greater levels. Analysis will be performed at a central lab and will be based on either the archival or fresh tissue sample.
5. Patients must have a formalin-fixed, paraffin embedded tumor specimen or unstained slides identified and available for analysis, to enable determination of the expression of B7-H3 within tumor specimens using IHC staining. If an archived tumor specimen is not available, patients who undergo a fresh tumor biopsy can have B7-H3 expression evaluated from the fresh sample and will not need to supply

a formalin-fixed, paraffin embedded tumor specimen. In cases where an archived sample and fresh tumor sample are both available, B7-H3 expression can be confirmed with either sample determined to be positive for B7-H3 staining, as determined by the sponsor.

6. With the exception of patients with non-measurable neuroblastoma (see criteria 3a above), patients must have measurable disease as per RECIST 1.1 criteria and documented by computed tomography (CT) and/or magnetic resonance imaging (MRI). Note: Lesions to be used as measurable disease for the purpose of response assessment must either a) not reside in a field that has been subjected to prior radiotherapy, or b) have demonstrated clear evidence of radiographic progression since the completion of prior radiotherapy and prior to study enrollment.
7. Karnofsky/Lansky ≥ 70
8. Life expectancy > 12 weeks

Laboratory Inclusion Criteria:

9. Acceptable laboratory parameters as follows:

a) Platelet count ≥ 75 x 10^3/microliter (≥ 75 x 10^9/L [international system of units: SI]) without transfusion within 2 weeks prior to the initiation of study drug

b) Hemoglobin that can be maintained at ≥ 8.0 g/dL (> 80 g/L [SI]) with transfusions

c) Absolute neutrophil count ≥ 0.75 x 10^3 /L [SI]) in the absence of any growth factor support within 2 weeks prior to the initiation of study drug

d) ALT and AST ≤ 3x ULN

e) Total bilirubin ≤ 1.5x ULN

f) Adequate renal function defined by serum creatinine based on age/gender as in the table below or a measured (by 24-hr urine collection or nuclear medicine scan) creatinine clearance ≥ 50 ml/min/1.73 m^2:

Threshold Creatinine Values for Children

Age	Maximum Serum Creatinine by mg/dL or µmol/L (SI)	
	**Male**	**Female**
1 to < 2 years	0.6 or 53.0	0.6 or 53.0
2 to < 6 years	0.8 or 70.7	0.8 or 70.7
6 to < 10 years	1 or 88.4	1 or 88.4
10 to < 13 years	1.2 or 106.1	1.2 or 106.1
13 to < 16 years	1.5 or 132.6	1.4 or 123.8
≥ 16 years	1.7 or 150.3	1.4 or 123.8

The threshold creatinine values in this table were derived from the Schwartz formula for estimating GFR **(29)** utilizing child length and stature data published by the CDC.

Reproductive Inclusion Criteria:

10. Female patients must not be pregnant. Female patients of childbearing potential must have a negative urine or serum pregnancy test performed within 72 hours prior to the initiation of study drug administration. Further, female patients of childbearing potential must agree to use highly effective contraceptive measures from the time of consent through 120 days after discontinuation of study drug administration. For female patients, two forms of contraception must be utilized and may include oral, transdermal, injectable or implantable contraceptives, intrauterine device, female condom, diaphragm with spermicide, cervical cap, use of a condom by the sexual partner or a sterile sexual partner. Periodic abstinence (e.g., calendar, ovulation, symptothermal and postovulation methods) and withdrawal are not considered acceptable forms of contraception in this study.
11. Male patients with partners of childbearing potential must use barrier contraception from initiation of the study drug to 120 days after discontinuation of the study drug. In addition, male patients should also have their partners use another method of contraception from the time of consent through 120 days after discontinuation of study drug administration.
12. Female patients must not be breastfeeding.

Previous Checkpoint Inhibitor Therapy:

13. Patients who have previously received an immune checkpoint inhibitor (e.g., anti-PD-L1, anti-PD-1, anti-CTLA-4) prior to enrollment must have any toxicities related to the checkpoint inhibitor resolved to Grade 1 or baseline (prior to the checkpoint inhibitor) to be eligible for enrollment.

This excludes patients who experienced the following immune checkpoint inhibitor-related AEs (i.e., the following AEs make the patient ineligible despite the AE resolving to Grade 1 or baseline):

a) ≥ Grade 3 ocular AE

b) Changes in liver function tests that me 3 x ULN of either ALT/AST with concurrent > 2x ULN of total bilirubin and without any alternate etiology)

c) ≥ Grade 3 neurologic toxicity

d) ≥ Grade 3 colitis

e) ≥ Grade 3 myocarditis, myositis, or autoimmune related neuromuscular toxicity such as myasthenia gravis

Exclusion Criteria:

1. Patients with a history of symptomatic central nervous system (CNS) metastases or symptomatic skull base lesions, are excluded, unless they have been treated and are asymptomatic, and they meet the following at the time of enrollment:

    a) No concurrent treatment for the CNS disease (e.g. surgery, radiation, corticosteroids ≥ 0.2mg/kg/day prednisone or equivalent)

    b) 14 days without progression of CNS metastases on MRI or CT after last day of treatment of previous therapy for the CNS metastases

    c) Patients with leptomeningeal disease or cord compression are excluded

2. Patients with any history of known or suspected autoimmune disease with the specific exceptions of vitiligo, resolved childhood atopic dermatitis, psoriasis not requiring systemic treatment within the past 2 years, laboratory testing. Patients with a history of asthma will NOT be excluded, provided the asthma is well controlled.

3. History of prior allogeneic bone marrow/stem cell or solid organ transplantation.

4. Patients receiving autologus stem cell transplantation must wait 8 weeks before initiation of study drug administration.

5. Patients receiving myelosuppressive chemotherapy must wait 4 weeks before initiation of study drug administration. Patients receiving other agents (e.g., biologics) must wait 2 weeks before initiation of study drug administration.

6. Patients receiving focal radiation therapy must wait 2 weeks prior to the initiation of study drug administration. Patients receiving large field radiation (e.g., whole lung, whole abdomen or pelvis) must wait 4 weeks prior to the initiation of study drug administration. Patients receiving ^{131}I-MIBG therapy must wait 6 weeks prior to the initiation of study drug administration.

7. Treatment with corticosteroids (0.2 mg/kg/day prednisone or equivalent) or other immune suppressive drugs within the 14 days prior to the initiation of study drug administration. Steroids for topical, ophthalmic, inhaled or nasal administration are allowed.

8. History of clinically significant cardiovascular disease, including but not limited to:

    a) Uncontrolled hypertension: systolic or diastolic blood pressure consistently > 1.5x the upper limit of normal for age

    b) QTcB prolongation > 480 msec

    c) A measured left ventricular fractional shortening of < 28%

9. Evidence of active viral, bacterial, or systemic fungal infection requiring parenteral treatment within 7 days prior to the initiation of study drug. Patients requiring any systemic antiviral, antifungal, or antibacterial therapy for active infection must have completed treatment no less than one week prior to the initiation of study drug. Patients on antimicrobial prophylaxis (e.g., for pneumocystis carinii infection), may continue on a stable dose of the antimicrobial for that purpose.

10. Known positive testing for human immunodeficiency virus or history of acquired immune deficiency syndrome.

11. Known history of hepatitis B or hepatitis C infection or known positive test for hepatitis B surface antigen, hepatitis B core antigen, or hepatitis C polymerase chain reaction.

12. Second primary invasive malignancy that has not been in remission for greater than 2 years. Exceptions that do not require a 2-year remission include: non-melanoma skin cancer; cervical carcinoma in situ on biopsy; or squamous intraepithelial lesion on PAP smear; or resected melanoma in situ.

13. History of severe trauma or major surgery within 4 weeks prior to the initiation of study drug administration.

14. Any serious underlying medical or psychiatric condition that, in the opinion of the Investigator, would impair the ability of the patient to receive or tolerate the planned treatment at the investigational site.

15. Known hypersensitivity to recombinant proteins, polysorbate 80 or any excipient contained in the drug formulation for MGA271.

16. Vaccination with any live virus vaccine within 4 weeks prior to the initiation of study drug administration. Inactivated annual influenza vaccination is allowed.

17. Employees or children of employees of MacroGenics, Inc., or of any investigative site staff directly involved with this study.

18. Any issue that, in the opinion of the Investigator, would contraindicate the study or confound the results of the study.
