# Supplementary material for: A Phase I, Open-Label, Dose Escalation Study of Enoblituzumab in Children and Young Adults with B7-H3–Expressing Relapsed or Refractory Solid Tumors
Source: Cancer Res Commun. 2025 Sep 10;5(9):1574–83. doi: 10.1158/2767-9764.CRC-25-0293 (PMC12421222; doi:10.1158/2767-9764.CRC-25-0293)
Supplement: Supplementary Data File 3 — Guidelines for the Management of Infusion-Related Reactions [file crc-25-0293_supplementary_data_file_3_suppsd3.pdf]

Guidelines for the Management of Infusion-Related Reactions

The following are treatment guidelines (which may be modified as needed by the responsible Investigator according to the best practices of medicine) for infusion reactions:

Grade 1:

- Slow the infusion rate by 50%.
- Monitor the patient for worsening of condition.
- Continue rate at 50% reduction and increase dose rate to the original rate by doubling the infusion rate after 30 minutes, as tolerated to the initial rate.
- If a patient has an infusion reaction with MGA271, prophylactic preinfusion medications should be given prior to all subsequent MGA271 infusions.
- With a Grade 1 infusion reaction of MGA271, the following prophylactic preinfusion medications are recommended prior to future infusions of MGA271:
    - diphenhydramine 0.5 mg/kg (maximum = 50 mg/dose) or equivalent
    - acetaminophen 10 mg/kg (maximum = 650 mg/dose) at least 30 minutes before additional study drug administrations.

Grade 2:

- Stop the infusion.
- Administer diphenhydramine hydrochloride 0.5 mg/kg IV (maximum = 50 mg/dose) or equivalent, acetaminophen 10 mg/kg orally (maximum = 650 mg/dose) for fever, and oxygen and bronchodilators for mild bronchospasm.
- Resume the infusion at 50% of the prior rate once the infusion reaction has resolved or decreased to Grade 1. The rate may then be escalated to the original rate after 30 minutes, as tolerated.
- Monitor for worsening condition. If symptoms recur, discontinue the infusion; no further study drug will be administered at that visit.
- If a patient has an infusion reaction with MGA271, prophylactic pre-infusion medications should be given prior to all subsequent MGA271 infusions.
- For patients with Grade 2 infusion reactions despite premedication with diphenhydramine and acetaminophen, corticosteroids (hydrocortisone 0.5 mg/kg IV (maximum = 50 mg/dose) or equivalent should be added

for acute management of the event and should be added to the premedication regimen for subsequent dosing of MGA271.

Grade 3:

- Stop the infusion and disconnect the infusion tubing from the patient.
- To avoid exacerbation of infusion reaction or CRS: do not flush the tubing - aspirate residual drug from the port lumen.
- Administer diphenhydramine hydrochloride 0.5 mg/kg IV (maximum = 50 mg/dose), hydrocortisone 1.0 mg/kg IV, (maximum = 100 mg/dose) or equivalent, and other medications/treatment as medically indicated. Higher doses of corticosteroids (e.g., methylprednisolone 1-2 mg/kg IV, dexamethasone 0.2 -0.5 mg/kg) may also be considered for acute management.
- IV fluids, supplemental oxygen and bronchodilators should be considered.

  If symptoms have resolved to baseline within 12 hours, a re-challenge may be considered at the next scheduled dose, with a 50% reduction of infusion rate. In addition, patients should be pre-medicated for this re-challenge and for any subsequent doses of MGA271 with the following: diphenhydramine hydrochloride 0.5 mg/kg IV (maximum = 50 mg/dose), oral acetaminophen 10 mg/kg (maximum = 650 mg/dose) and hydrocortisone 0.5 mg/kg IV (maximum = 100 mg/dose). Patients who experience a second Grade 3 infusion reaction at the time of re-challenge of MGA271 (irrespective of duration), should not receive further MGA271. Patients who have a Grade 3 infusion reaction that does not resolve within 12 hours despite medical management should not receive further MGA271 treatment.
- Report as an Immediately Reportable Event (IRE) within 24 hours.
- Report the event as an SAE, if appropriate.

Grade 4:

- Stop the infusion and disconnect the infusion tubing from the patient.
- To avoid exacerbation of infusion reaction or CRS: do not flush the tubing - aspirate residual drug from the port lumen.
- Administer diphenhydramine hydrochloride 0.5 mg/kg IV (maximum dose = 50 mg/dose), methylprednisolone 2 mg/kg IV (or more as considered appropriate), and other medications/treatment as medically indicated (e.g., an IL-6 receptor inhibitor or IL-6 inhibitor,

an IL-2 receptor inhibitor, and/or an anti- TNFa antibody).

- Give epinephrine or bronchodilators as indicated.
- Support ventilation and blood pressure as indicated.
- Report as an IRE within 24 hours.
- Report the event as an SAE within 24 hours.
- Patients who have a grade 4 infusion reaction should not receive further MGA271.
