# Supplementary material for: A Phase I, Open-Label, Dose Escalation Study of Enoblituzumab in Children and Young Adults with B7-H3–Expressing Relapsed or Refractory Solid Tumors
Source: Cancer Res Commun. 2025 Sep 10;5(9):1574–83. doi: 10.1158/2767-9764.CRC-25-0293 (PMC12421222; doi:10.1158/2767-9764.CRC-25-0293)
Supplement: Supplementary Table 1 — B7-H3 Expression Patterns for Cancer Cells (CC) and Tumor Vasculature (VAS) [file crc-25-0293_supplementary_table_1_suppst1.pdf]

Table S1. B7-H3 Expression Patterns for Cancer Cells (CC) and Tumor Vasculature (VAS)

		% of Cancer Cells and Tumor Vasculature Staining at Different Levels							
Patient	Diagnosis	CC 0	CC 1+	CC 2+	CC 3+	VAS 0	VAS 1+	VAS 2+	VAS 3+
1	HCC	0	0	100	0	0	0	0	100
2	NBNM	0	0	30	70	0	80	20	0
3	NBNM	5	5	20	70	0	0	0	100
4	NBNM	0	30	60	10	0	20	70	10
5	NBNM	1	0	49	50	50	50	0	0
6	Liver sarcoma	0	10	50	40	0	10	70	20
7	NBNM	30	30	30	10	0	80	20	0
8	NBNM	0	10	0	90	0	90	10	0
9	NB	0	0	0	100	NE*	NE*	NE*	NE*
10	NBNM	0	0	10	90	0	80	20	10
11	RMS	10	80	10	0	0	20	70	0
12	RMS	10	20	10	60	30	30	30	10
13	OS	10	0	0	90	30	10	0	60
14	OS	0	10	40	50	0	20	80	0
15	OS	0	10	30	60	0	80	20	0
16	OS	10	70	10	10	45	50	0	5
17	OS	5	40	50	5	O	O	40	60
18	OS	20	70	10	0	30	60	10	0
19	DSRCT	0	20	70	10	30	70	0	0
20	Melanoma	0	10	60	30	20	30	40	10
21	USRBCT	0	10	50	40	20	30	40	10
22	Synovial sarcoma	0	20	75	5	0	50	50	0
23	DSRCT	0	20	70	10	0	30	70	10
24	Ewing	30	30	20	20	0	40	50	10
Average		5	21	36	38	11	40	31	18

*NE = not evaluable

HCC = hepatocellular carcinoma; NBNM = neuroblastoma with non-measurable disease; NB = neuroblastoma; RMS = rhabdomyosarcoma; OS = osteosarcoma; DSRCT = desmoplastic small round cell tumor; USRBCT = undifferentiated small round blue cell tumor
