# Supplementary material for: A Phase I, Open-Label, Dose Escalation Study of Enoblituzumab in Children and Young Adults with B7-H3–Expressing Relapsed or Refractory Solid Tumors
Source: Cancer Res Commun. 2025 Sep 10;5(9):1574–83. doi: 10.1158/2767-9764.CRC-25-0293 (PMC12421222; doi:10.1158/2767-9764.CRC-25-0293)
Supplement: Supplementary Table 2 — Average B7-H3 Expression Patterns for Treated Patients by Diagnosis [file crc-25-0293_supplementary_table_2_suppst2.pdf]

Table S2. Average B7-H3 Expression Patterns for Treated Patients by Diagnosis

		% of Cancer Cells and Tumor Vasculature Staining at Different Levels							
Diagnosis	n	CC 0	CC 1+	CC 2+	CC 3+	VAS 0	VAS 1+	VAS 2+	VAS 3+
Neuroblastoma	8	5	9	25	61	7	57	20	16
Osteosarcoma	6	8	33	23	35	18	37	25	23
Rhabdomyosarcoma	2	10	50	10	30	15	30	50	5
Other sarcoma	3	10	20	48	22	0	33	57	10
DSRCT	2	0	20	70	10	15	50	35	0

DSRCT = desmoplastic small round cell tumor
