# Supplementary material for: A Phase I, Open-Label, Dose Escalation Study of Enoblituzumab in Children and Young Adults with B7-H3–Expressing Relapsed or Refractory Solid Tumors
Source: Cancer Res Commun. 2025 Sep 10;5(9):1574–83. doi: 10.1158/2767-9764.CRC-25-0293 (PMC12421222; doi:10.1158/2767-9764.CRC-25-0293)
Supplement: Supplementary Table 3 — Parameter Estimates for Final Pharmacokinetic Model [file crc-25-0293_supplementary_table_3_suppst3.pdf]

Table S3. Parameter Estimates for Final Pharmacokinetic Model

	Fitted Parameters Mean (%SE)	Parameters from Fitted Bootstrap Median (95% CI)
CL (mL/hr)	1.85 (40.2)	1.80 (1.01 - 3.59)
V_1 (L/m^2)	1.59 (4.3)	1.57 (1.44 – 1.84)
Q (mL/hr)	24.3 (9.7)	24.7 (20.6 – 34.3)
V_2 (L)	2.43 (7.0)	2.44 (2.01 – 3.23)
IIV on CL (%)	169 (22.0)	165 (91.4 – 232)
IIV on V_1 (%)	29.6 (15.8)	28.8 (8.7 – 46.6)
Residual Error (%)	22.0 (4.4)	21.2 (13.7 – 32.6)

CI = Confidence interval; PK parameters exclude one outlier subject.
