# Supplementary material for: A Phase I, Open-Label, Dose Escalation Study of Enoblituzumab in Children and Young Adults with B7-H3–Expressing Relapsed or Refractory Solid Tumors
Source: Cancer Res Commun. 2025 Sep 10;5(9):1574–83. doi: 10.1158/2767-9764.CRC-25-0293 (PMC12421222; doi:10.1158/2767-9764.CRC-25-0293)
Supplement: Supplementary Table 4 — Representativeness of Study Participants [file crc-25-0293_supplementary_table_4_suppst4.pdf]

Table S4. Representativeness of Study Participants

Cancer type(s)/subtype(s)/stage(s)/condition	Relapsed, metastatic pediatric solid tumors (e.g. neuroblastoma and osteosarcoma - most common diagnoses of patients enrolled in this trial)
Considerations related to:	
Sex	Both neuroblastoma and osteosarcoma are slightly more common in boys with a male:female incidence of incidence of 1.1 – 1.3:1.
Age	Neuroblastoma is typically a disease of young children with the highest incidence rate seen in the 1st year of life and 90% of cases occurring before the age of 5. Osteosarcoma has a bimodal distribution with the largest peak occurring in adolescents and young adults (10 – 24 years), and a smaller peak occurring after age 60.
Race/ethnicity	In the U.S. neuroblastoma has a higher incidence among whites (12.8 per million), compared to Hispanics (9.9 per million) or blacks (9.6 per million). In adolescents and young adults osteosarcoma is slightly more common in blacks and Hispanics (incidence rate 7.9) compared to whites (incidence rate 6.8)
Geography	In the U.S. there are 600 - 800 cases of neuroblastoma diagnosed each year. Approximately 45% of these cases are considered to be “high-risk”, and there are ~ 150 deaths from neuroblastoma in the U.S. each year. There are approximately 400 - 500 cases of osteosarcoma diagnosed in children and young adults in the U.S. each year. The disease is metastatic at diagnosis in ~ 20% of cases. Approximately 140 children and young adults die from osteosarcoma each year in the U.S.
Other considerations	Other solid tumors represented in this study included Ewing sarcoma, melanoma, rhabdomyosarcoma, hepatocellular carcinoma, desmoplastic small round cell tumor, synovial sarcoma and undifferentiated sarcoma. The small number of patients with

	these diagnoses preclude drawing any conclusions about representativeness.
Overall representativeness of this study	Neuroblastoma: The median age of the neuroblastoma patients participating in this trial was 10 years. This somewhat older age likely reflects the fact that these were children with relapsed disease who had already failed multiple prior therapies. The male to female ratio was 2:1, which is higher than the expected ratio of 1.1 -1.3:1. Seven of the eight patients were white.<br>Osteosarcoma: The median age of the osteosarcoma patients was 16.5 years. The male to female ratio was 2:1, again somewhat higher than the expected ratio of 1.1 – 1.3:1. There were 4 white, 1 Hispanic, and 1 patient of unknown race enrolled in the trial.
